# Racial Bias in National Football League Officiating

**DOI:** 10.3389/fsoc.2020.00048

**Published:** 2020-07-14

**Authors:** Dawson G. Eiserloh, Jeremy J. Foreman, Elizabeth C. Heintz

**Affiliations:** School of Kinesiology, University of Louisiana at Lafayette, Lafayette, LA, United States

**Keywords:** African-American, discrimination, NFL, official, penalties, race, referee, umpire

## Abstract

Racial bias in sport is a prevalent research topic. Much of the previous research regarding bias among referees in sport focused on sports such as baseball, basketball, hockey, and soccer. Professional American football is unique because race is more clearly defined when compared to these other sports. Additionally, by examining holding penalties, which are known to be more subjective and called predominately by a single official on the field (i.e., the umpire), racial bias in officiating can be more efficiently analyzed in professional American football. The purpose of this study is to examine potential racial bias regarding holding penalties in the National Football League (NFL). Three years of data from the 2013 to 2014 through 2015 to 2016 NFL seasons were used, including the races of officials and players involved in holding penalties. Results showed no evidence of racial bias in the calling of holding penalties by White officials. However, Black umpires were found to call more holding penalties when led by a White referee. Additionally, Black players were more likely to have holding penalties called on them earlier in the game by all officials.

## Racial Bias Among National Football League Referees

Bias in sports is a prevalent research topic and scholars have examined potential biases in sport in a variety of contexts (e.g., Pope et al., 2018; Salaga and Juravich, 2020). One context bias has been analyzed in sport is the potential bias of sports officials. Officials may make biased decisions against certain teams (Rodenberg and Lim, 2009; Rodenberg, 2011) or based on certain personal attributes of players (Caudill et al., 2014; Gift and Rodenberg, 2014). While some biases may be based on team (Lago-Peñas and Gómez-López, 2016) or individual performance (Caudill et al., 2014), others may be based on demographic characteristics, such as race (Price and Wolfers, 2010; Hamrick and Rasp, 2015; Tainsky et al., 2015).

Biases based on race (i.e., racial bias) continues to be a necessary discussion topic in sports (e.g., Salaga and Juravich, 2020; Foreman and Turick, in press). While racial bias in sports is typically investigated in the hiring and opportunities of players, coaches, and management (e.g., Ducking et al., 2015; Foreman et al., 2018), racial bias may exist in officiating as well. However, in studies examining racial bias in officiating, race has not always been clearly defined (e.g., Price and Wolfers, 2010). This difficulty defining race may be a result of globalized sports attracting a wide variety of people across a broad spectrum of races, ethnicities, nationalities, and cultures. Indeed, previous research on bias among referees related to demographic characteristics focused largely on professional baseball, basketball, hockey, and soccer (Price and Wolfers, 2010; Mongeon and Longley, 2015; Tainsky et al., 2015). However, race is more clearly defined in American football than in other major professional team sports (Foreman et al., 2018).

Therefore, the purpose of this study is to determine if racial bias is present in penalty calls made by referees in the National Football League (NFL). This study builds on previous research regarding biases of sports officials, which are presented next in the literature review section. Following the literature review section is a brief section about the empirical setting of the NFL, and then the methods section reveals how the penalty data from the 2013 to 2014 through 2015 to 2016 NFL seasons were collected and analyzed. After the methods are explained, the results are presented, which indicate that Black officials call more penalties against all players when led by a White official. Finally, the results are explained in more detail within the discussion section and a summary of findings, as well as limitations and opportunities for future research, are presented in the conclusion section.

## Literature Review

Several studies examined potential bias among referees that was not based on racial bias (e.g., bias against certain teams). For example, in a study on the Dallas Mavericks, Rodenberg and Lim (2009) found no distinct biases during the regular season, although bias was found from one referee during playoff games. Rodenberg (2011) later found that there was no evidence of bias against the Miami Heat when investigating two specific referees over a 9-year period. Further, no bias was found toward losing teams or home teams (Deutscher, 2015). Contrarily, soccer officials have been found to favor higher-level teams and determine extra time in matches based on game score (Lago-Peñas and Gómez-López, 2016), Constantinou et al. (2014) found evidence of referees being biased toward certain home teams, and Corrigan et al. (2019) found that officials are less likely to make calls that will directly impact game score.

There is also evidence bias official decisions based on personal attributes other than race or similar cultural or demographic characteristics. Gift and Rodenberg (2014) found that shorter NBA referees officiate games differently and tend to call more fouls than taller referees. It is also suggested that star NBA players receive preferential treatment toward the end of playoff games (Caudill et al., 2014); however, Deutscher (2015) found no evidence bias among officials when examining at a variety of factors, including star players.

Evidence of racial bias among officials also exists. Racial bias is important to understand, in part, because it can lead to unfair consequences (Kelly and Roedder, 2008). Racial bias can be and has been examined through the lens of racial profiling, whereby people are identified based on their race and exposed to unfair consequences (e.g., police searches or criminal charges; Gabor, 2004; Hernández-Murillo and Knowles, 2004). Similar to police searches and criminal charges, a more transparent setting to analyze racial bias in society is in sport (Kelly and Roedder, 2008; Price and Wolfers, 2010), where penalties are similar to minor workplace deviance (Foreman et al., 2019a,b; Ford et al., in press).

Pope and Pope (2015) found that referees, regardless of experience, were biased in favor of players from their native country. Price and Wolfers (2010, 2012) reported that more personal fouls were called against players when opposite-race crews were officiating compared to when own-race crews were officiating, showing that referees tend to call more fouls on players of another race and disputing claims from the NBA that referees did not show bias. Likewise, Mongeon and Longley (2015) found evidence that English players get more penalties called on them by French and American referees in the NHL. However, mixed results of racial discrimination exist regarding Major League Baseball (MLB) strike-ball calls made by umpires (Hamrick and Rasp, 2015; Tainsky et al., 2015).

Race is easier to define in American football compared to other sports such as baseball, basketball, hockey, or soccer (Foreman et al., 2018) where racial bias in officiating has previously been examined. Andrew (2017) presents evidence of racial bias in American football, showing that more penalties are called against historically Black universities than against predominantly White universities; however, racial bias in American football has not been investigated at the professional level or against individuals. Therefore, we present the following research question:

Is there a relationship between the races of officials and players in the NFL and the propensity of a penalty being called?

## Empirical Setting

The NFL was created in 1920, and currently has 32 teams. To ensure fairness in competition among each of the teams, rules, and penalties continued to be established throughout the existence of the league (Foreman et al., submitted). To identify penalties within the course of a game, seven officials monitor various aspects of gameplay (NFL Operations, 2020b). While NFL penalties have been examined by several scholars (Foreman et al., submitted), another topic of interest among scholars is racial bias in the NFL (e.g., Collins, 2007; Foreman et al., 2018). For example, the NFL has received substantial attention in the media and among scholars for potential racial bias in head coach hiring practices (Duru, 2007; Foreman and Turick, in press). Given the attention the NFL has received regarding potential racial biases, it seems possible that racial bias may also be occurring among league referees when identifying (or “calling”) penalties, such as holding penalties.

## Methods

### Aims and Objectives

To examine potential racial bias in penalties called in professional American football, the NFL is used as an empirical setting in which penalties called against individual players could be studied based on the race of players and officials.

### Research Questions

Given that officials may possess biases against races other than their own or against a certain race, regardless of their own race, the primary research question guiding this study is: Is there a relationship between the races of officials and players in the NFL and the propensity of a penalty being called? To examine the effects of official and player races on holding penalties called, two analyses are used: a player-level analysis and a game-level analysis.

### Data Collection and Sample

The sample consists of penalty data from the 2013 to 2014 through 2015 to 2016 NFL seasons. Data on penalties were collected from play-by-play information from *pro-football-reference.com* (Sports Reference, 2020). Specifically, the present study focuses on offensive holding penalties due to their inherent subjectivity. Offensive holding is perceived as a judgement call (Schalter, 2012; Allentuck, 2019), and therefore, would likely allow for more bias than non-judgement calls. Offensive holding occurs when a player attempts to restrict a defender by “grabbing or tackling an opponent; hooking, jerking, twisting, or turning him; or pulling him to the ground” (NFL Operations, 2020a, p. 1). American football is a physical game (Foreman et al., submitted), and these maneuvers could be described as happening on every play (Allentuck, 2019). Therefore, offensive holding penalties can be useful for analyzing bias among referees because it requires a degree of judgement by the referee to determine whether the violation occurred.

Offensive holding penalties in the NFL are also advantageous to study for racial bias because calling offensive holding penalties on players is primarily the duty of a single official: the umpire (NFL Operations, 2020b). While one of the umpire's general responsibilities is to look for potential holding calls, the referee is the crew leader of the officials and has the authority to rule on disputed calls and can change or make calls at any time (NFL Operations, 2020b). Therefore, data on umpires and referees for every regular season game in the three-season sample period were also collected from *pro-football-reference.com* (Sports Reference, 2020).

### Dependent Variables

There are two analyses used: a player-level analysis and a game-level analysis. The dependent variable for the player-level analysis holds one of four values representing four possible outcomes. Each outcome is a dichotomous variable and they are indicative of (a) whether a Black umpire called an offensive holding penalty on a Black player (*BUMPBPLAYER*), (b) whether a Black umpire called an offensive holding penalty on a White player (*BUMPWPLAYER*), (c) whether a White umpire called an offensive holding penalty on a Black player (*WUPMBPLAYER*), or (d) whether a White umpire called an offensive holding penalty on a White player (*WUMPWPLAYER*). *WUMPWPLAYER* is used as the base outcome. The dependent variable in the game-level analysis is the percentage of holding penalties called on Black players per game (*BLACKPENS*).

### Independent Variables

The independent variable in the player-level analysis is a binary variable showing whether the referee is White (*WHITEREF*). *WHITEREF* is coded “1” for White referees and “0” for non-White referees. The independent variables in the game-level analysis are binary variables that indicate whether the referee is White (*WHITEREF*), umpire is White (*WHITEUMP*), or both the referee and umpire are White (*WHITEUMP* × *WHITEREF*).

### Control Variables

Control variables for the player-level analysis consist of dichotomous variables indicative of whether the penalty was called on the home team (*HOMETEAM*) and losing team (*LOSINGTEAM*), as well as continuous variables representing the absolute value of the score difference between teams (*SCOREDIFF*) and how many minutes were left in the game when the penalty was called (*TIMELEFT*). These are variables that have the potential to affect the likelihood of a penalty being called.

### Data Analysis

For the player-level analysis, due to the four possible outcomes that cannot be ordered, a multinomial logistic regression analysis is used to examine the effect of several determinants on the dependent variable. For the game-level analysis, given the continuous nature of the dependent variable, a linear regression is used to estimate the effects of the independent variables.

## Results

Summary statistics for the player-level and game-level analyses are shown in Tables 1, 2, respectively. Table 1 shows player-level summary statistics (*N* = 1,628). Black umpires called offensive holding penalties on Black players in 17% of the observation and on White players in 16.5% of the observations. White umpires called offensive holding penalties on Black players in 34.1% of the observations and on White players in 32.5% of the observations.

**Table 1:** Player-level summary statistics (*N* = 1,628).

**Variable**	**Mean**	**Std. Dev**.	**Min**	**Max**
BUMPBPLAYER	0.170	0.375	0	1
BUMPWPLAYER	0.165	0.371	0	1
WUMPBPLAYER	0.341	0.474	0	1
WUMPWPLAYER	0.325	0.468	0	1
HOMETEAM	0.514	0.500	0	1
LOSINGTEAM	0.448	0.497	0	1
SCOREDIFF	7.813	7.045	0	41
TIMELEFT	28.204	15.780	0	60
WHITEREF	0.850	0.357	0	1

**Table 2:** Game-level summary statistics (*N* = 657).

**Variable**	**Mean**	**Std. Dev**.	**Min**	**Max**
BLACKPENS	0.507	0.385	0	1
WHITEUMP	0.677	0.468	0	1
WHITEREF	0.868	0.339	0	1

Game-level summary statistics are reported in Table 2 (*N* = 657). The average percent of penalties called on Black players per game was 50.7%. White umpires officiated 67.7% of the games in the sample and White referees officiated 86.8% of the games in the sample.

Table 3 reports the results from the player-level multinomial logistic regression. The variable *WHITEREF* was statistically significant (*p* < 0.01) for penalties called by a Black umpire on a Black player and for penalties called by a Black umpire on a White player. *WHITEREF* was not statistically significant for penalties called by a White umpire on a Black player. The variable *TIMELEFT* was statistically significant for penalties called by a Black umpire on a Black player (*p* < 0.01), for penalties called by a Black umpire on a White player (*p* < 0.01), and for penalties called by a White umpire on a Black player (*p* < 0.01). Table 4 reports results from the game-level linear regression. None of the independent variables were statistically significant in the game-level analysis.

**Table 3:** Player-level multinomial logistic regression.

**Variable**	**Black umpire and player**		**Black umpire and white player**		**White umpire and black player**	
	**Coefficient**	**Std. Err**.	**Coefficient**	**Std. Err**.	**Coefficient**	**Std. Err**.
WHITEREF	1.362^**^	0.285	1.397^**^	0.288	−0.032	0.154
HOMETEAM	0.157	0.151	0.006	0.152	0.067	0.123
LOSINGTEAM	−0.174	0.156	0.208	0.156	0.007	0.126
SCOREDIFF	0.007	0.011	−0.014	0.012	0.004	0.009
TIMELEFT	0.021^**^	0.005	0.01	0.005	0.016^**^	0.004
Constant	−2.500^**^	0.362	−2.166^**^	0.348	−0.443^*^	0.220

WUMPWPLAYER is the base outcome.

* p < 0.05,

** *p < 0.01, two-tailed*.

**Table 4:** Game-level linear regression.

**Variable**	**Coefficient**	**Std. Err**.
WHITEUMP	−0.020	0.115
WHITEREF	−0.041	0.111
WHITEUMP × WHITEREF	0.049	0.120
Constant	0.528^**^	0.107

** *p < 0.01, two-tailed*.

## Discussion

The purpose of this study was to examine whether racial bias exists in the calling of NFL offensive holding penalties. This study found no evidence of racial bias in the direct calling of holding penalties by White officials. While this result seems to be inconsistent with other evidence of racial bias in officiating (e.g., Price and Wolfers, 2010, 2012; Deutscher, 2015; Mongeon and Longley, 2015), the racial bias either is less prevalent in the NFL or it may be taking other forms. If there is less racial bias in the NFL, relative to other professional North American team sports (e.g., NBA, NHL), there may be several reasons why this is occurring.

Price and Wolfers (2010, 2012) found evidence of racial bias in NBA officiating. Racial bias in NBA penalty calls could be due to increased visibility of a player's race, as faces and skin are more visible in basketball than in American football. Increased visibility of an athlete's race could lead to more opportunities for bias to occur. Therefore, the results of the present study appear to make sense in comparison to the findings by Price and Wolfers (2010, 2012).

Results of the present study also differ from those of Mongeon and Longley (2015), who found that English Canadian players had more penalties called on them by French Canadian referees than by English Canadian referees in the NHL. However, Mongeon and Longley reasoned that the result may be less based on ethnicity or language than it is the result of more aggressive playing styles of English Canadians (Mongeon and Longley, 2015). This could also be true for our results, because if Black and White players have similar playing styles, it would be more difficult to show bias. However, it is also worth noting that hockey and American football players wear similar amounts of gear, making the features of players in both sports difficult to see in competition and potentially making it more difficult for referees to exhibit bias. Therefore, it is interesting that there is evidence of bias in penalties called in hockey, but not in American football.

Another interesting finding of the present study is that Black umpires called more penalties on both Black and White players when working with White referees. Therefore, the findings within the present study may be less indicative of racial bias being less prevalent in the NFL and more indicative of racial bias being exhibited in other ways. More specifically, this finding may be indicative of increased pressure on Black umpires to call more penalties when the referee is White.

This conclusion is consistent with a similar conclusion presented by Foreman and Turick (in press) that minority head coaches feel more pressure than White coaches when making personnel decisions. Therefore, consistent with Foreman and Turick's suggestion that decisions of minority and White coaches will become more similar as minorities gain legitimacy and subsequent fears of adverse personnel decisions (e.g., dismissal) reduce, the amount of penalties Black umpires call when working with White referees may decrease to be similar to the amount they call when working with Black referees. This increased legitimacy may come when more Black officials are assigned the role of referee, because <14% of games in the sample were officiated by a Black referee.

Results of this study also indicate that Black players are more likely to get penalty calls earlier in the game than later in the game, regardless of the race of the officials. This is an interesting finding that could be occurring for several reasons. First, officials may be less likely to face scrutiny for calls made earlier in the game than later in the game. Calls made at the beginning of the game may not be thought to impact the outcome of a game as much as calls made toward the end of a game. Therefore, officials could feel less pressure to make the correct call at the beginning of a game. Further, if Black umpires do feel pressure to call more penalties on players, they may call them earlier in a game, knowing they will face less scrutiny than if the calls were made later in a game.

Another possible reason for referees making more calls against Black players at the beginning of a game is that referees do not have the same opportunities to warm up at the beginning of games, like players do. While players practice before a game begins, referees do not have the chance to practice making calls in a live setting before a game starts, which could lead to more erroneous calls based on biases earlier in the game. Without the opportunity to warm up at the beginning of the game, and without many delays that typically occur toward the ends of games, decisions made by officials at the beginning of games may be made with less time to think. Previous research found that decisions made by both Black and White Americans under severe time constraints are susceptible to racial bias against Black people (Payne, 2006). Therefore, the effect found in the present study, where both Black and White umpires are more likely to call penalties on Black players earlier in games is consistent with previous research on racial bias.

## Conclusion

This study did not find evidence of racial bias in the calling of holding penalties directly by White officials in the NFL. These results offer hope for overcoming racial bias in sport. However, Black umpires are more likely to call penalties when led by a White referee, even though they call fewer penalties, in general, than White umpires. These findings could be indicative of increased pressure on Black umpires when the referee is White. Additionally, Black players are more likely to have holding penalties called against them earlier in the game, regardless of the race of the officials.

### Limitations

While our results show that Black umpires are more likely to call penalties with a White referee and that Black players are more likely to have penalties called on them at the beginning of a game, we cannot definitively determine why these phenomena are occurring–only the statistical trends. Additionally, the use of penalty data within this study does not ensure whether penalties were called accurately.

### Future Research

Future research on this topic could investigate why Black umpires tend to call more penalties under a White referee and why Black players have more penalties called on them earlier in a game. Examining pressure felt by officials at different times in a game and under different referees, as well as investigating playing styles of Black and White players in American football at different points in a game, could provide additional insight into these findings. Since Black players are more likely to play peripheral (e.g., tackle), rather than interior (e.g., center or guard) positions (Foreman and Turick, in press), future research could also examine whether peripheral positions are easier for officials to see, and therefore, easier to penalize than interior positions, thus affecting the amount of penalties called on White and Black players in the NFL.

## Data Availability Statement

The datasets generated for this study are available on request to the corresponding author.

## Author Contributions

DE: wrote first draft of paper, collected data, and assisted with revisions. JF: collected and analyzed data, guided research process, edited the paper, and assisted with revisions. EH: revised first draft of manuscript and assisted with revisions. All authors contributed to the article and approved the submitted version.

## Conflict of Interest

The authors declare that the research was conducted in the absence of any commercial or financial relationships that could be construed as a potential conflict of interest.
